# Tailored Interventional Approaches to the Management of True and False Aneurysms Affecting Aberrant Visceral Arteries Are Associated with Enhanced Clinical Outcomes

**DOI:** 10.3390/jpm16030165

**Published:** 2026-03-16

**Authors:** Ottavia Borghese, Arisa Ibrahimi, Antonio Luparelli, Giulia Piermarini, Yamume Tshomba

**Affiliations:** 1 Unit of Vascular Surgery, Fondazione Policlinico Universitario A. Gemelli IRCCS, 00168 Rome, Italy; antonio.luparelli@guest.policlinicogemelli.it (A.L.); giuliapiermarini@unicatt.it (G.P.); 2 Unit of Vascular Surgery, Department of Cardiovascular Sciences, Università Cattolica del Sacro Cuore, 20123 Rome, Italy; arisa.ibrahimi01@icatt.it

**Keywords:** aberrant visceral artery, aneurysm of visceral arteries, visceral arteries pseudoaneurysm, complication

## Abstract

**Background:** Anatomical variations in visceral arteries are not so uncommon (up to 20% of cases in general population), with splenic and hepatic artery anomalies being the most frequently reported. Aberrant arteries may be affected with aneurysmal lesions that are rare but potentially fatal conditions. In their treatment, a comprehensive understanding and knowledge of the underlining anatomical variation are pivotal to prevent potential ischemic complications for the end organ. **Methods:** A comprehensive literature search on the PubMed, Cochrane and Scopus databases was done using the terms: “anomalous visceral artery aneurysm”, “Aberrant visceral arteries”, and “anomalous origin visceral vessels”. Eligible studies published from inception to 30 June 2024 were identified. Only those that had included the adopted treatment strategies (open, endovascular or hybrid repair) and the related outcomes (mortality, bleeding, end-organ ischemia, lesions of the surrounding organ, need for reintervention) were analyzed to evaluate the safety and efficacy of each approach. A narrative analysis of the indications informing the selection of each interventional treatment, based on individual procedural risks, was also presented. **Results:** A total of 30 publications describing 36 patients (mean age 48.9 ± 12.8 years, range 22–73 years) with aneurysms involving aberrant visceral arteries were included. Most patients were female (25/36, 69.4%). True aneurysms predominated (with a mean size of 30.5 ± 11.5 mm, range 6–60 mm), being reported in 33/36 (91.7%) patients. Most lesions involved a splenic artery arising from the superior mesenteric artery (27/36, 75.0%). Overall, 26/36 (72.2%) patients were symptomatic upon presentation, most commonly with abdominal or epigastric pain, often associated with nausea or vomiting, back pain or shortness of breath. All patients underwent preoperative Computed angiotomography or subtraction angiography to define the operative strategy. Most cases were managed electively (31/36, 86.1%), but 11.1% (4/36) of cases required urgent intervention (in one case the urgency status was not specified). Overall, 19/36 (52.8%) patients underwent purely endovascular repair, 15/36 (41.7%) were treated with open surgery, and 2/36 (5.6%) had hybrid procedures combining endovascular coiling with laparoscopic splenic artery ligation. Indication for treatment was based on vessel tortuosity, landing zones, and the presence of side branches supplying end organs. Early outcomes were favorable regardless of treatment strategies. A single organ-related complication was reported (1/36, 2.8%) following open/endovascular repair, consisting of mild pancreatitis, which resolved with conservative management. No perioperative or aneurysm-related deaths were reported in any of the included cases. No recurrent aneurysms or late aneurysm-related complications were described during the reported follow-up intervals (mean ≈ 10.5 months, range 1.5–42 months). **Conclusions:** Aneurysms arising from aberrant visceral arteries present unique challenges because their origin, course, and collateral networks deviate from standard anatomy. Patient selection and detailed anatomic mapping preoperatively are decisive as inadequate imaging or failure to recognize an aberrant origin can lead to the incomplete exclusion or inadvertent sacrifice of critical branches. Understanding the anatomy of visceral arteries and their variations is paramount in clinical practice, particularly when planning interventions for minimizing procedural risks, optimizing outcomes, and preventing potential complications. Contemporary practice favors endovascular repair due to lower perioperative morbidity, but success depends on vessel tortuosity, landing zones, and the presence of important side branches that supply end organs.

## 1. Introduction

Anatomical variation in visceral arteries encompasses a wide spectrum of configurations, including anomalous origins, the presence of additional branches or variations in branching angles and course [1].

An anomalous configuration of the visceral arteries is reported in approximately 20% of the general population, with the most common variant being a right hepatic artery from the superior. In about 5% of cases arising directly from the mesenteric artery, the left gastric artery instead originates directly from the aorta, whereas a splenic artery arising directly from the aorta is far less frequent, occurring in roughly 1% of cases [2].

The most frequently encountered lesions of aberrant visceral arteries are aneurysms that may be acquired (false aneurysm or pseudoaneurysm following trauma or infection), congenital or atherosclerotic-related conditions (true aneurysm) [3,4]. Despite being rare, pseudoaneurysms are at high risk of rupture with potentially life-threatening complications and should always be interventionally treated. Conversely, current indications for true aneurysm repair are based on size, the rate of growth or the individual risk factors for rupture (women of child-bearing age, collagenous disease, liver transplantation or portal hypertension) [4].

When an interventional management of such lesions is indicated with both surgical (artery ligation with or without revascularization or end-organ resection) and endovascular (embolization and/or stent graft positioning) techniques, a comprehensive understanding and knowledge of the underlining anatomical variation are pivotal to prevent potential complications [3,5,6]. Indeed, the efficacious and safe management of such lesions relay on the capacity to anticipate potential anatomical complexities to mitigate procedural risks.

In the present paper, we describe a comprehensive review of the current literature on reported cases of aneurysms and pseudoaneurysms affecting aberrant visceral arteries. A narrative analysis of the indications informing the selection of each interventional treatment, based on individual procedural risks, is also presented to instruction physicians and guide future management.

## 2. Methods

### 2.1. Study Selection, Data Extraction and Processing

This is a systematic literature review performed through the Medline (https://pubmed.ncbi.nlm.nih.gov/, U.S. National Library of Medicine, National Institute of Health), Scopus (Elsevier BV, Amsterdam, The Netherlands) and Cochrane databases from inception to 30 June 2024.

The free-text search terms “visceral artery,” “aneurysm,” “pseudoaneurysm,” “aberrant visceral arteries,” “endovascular”, “Open repair”, “Mortality”, “complications” and synonyms were used in combination with the Boolean operators “AND” and “OR” (Table S1).

**Figure 1:**
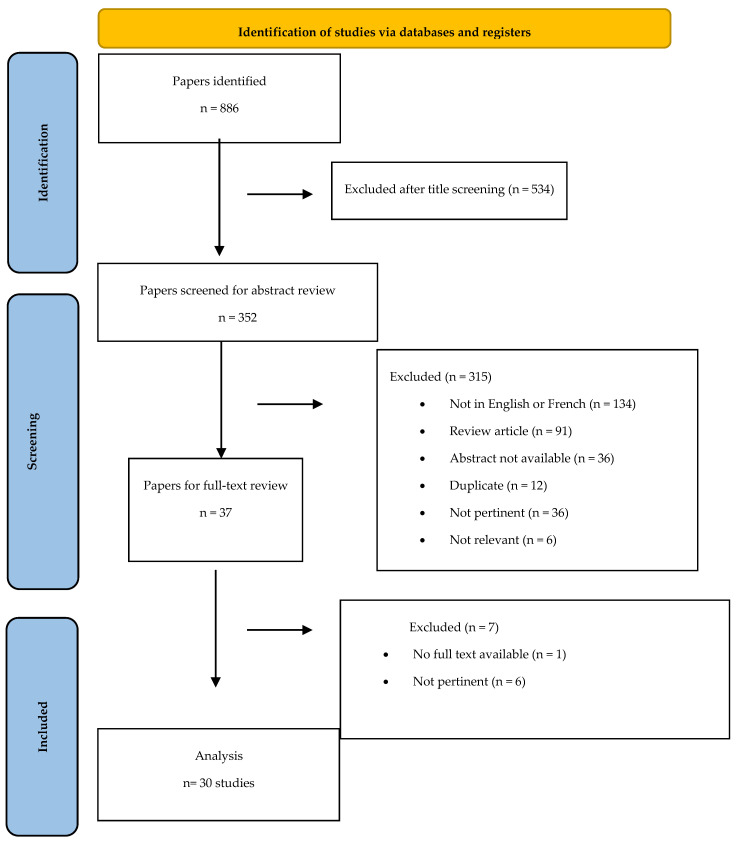
PRISMA flowchart. PubMed.gov, U.S. National Library of Medicine, National Institute of Health. From [8].

The Population, Intervention, Comparison and Outcome (PICO) model was used to frame the question of this systematic review (Table S2). To ensure systematic review quality, the Preferred Reporting Items for Systematic Reviews and Meta-Analyses (PRISMA) checklist and flow diagram were used [7,8] (Figure 1).

The studies were selected following title and abstract screening, and all duplicates were removed manually. Only studies written in the English or French language and about adult patients were included.

A full-text assessment was done and data entered independently by the same two reviewers (A.L. and G.P.) in a predefined database using Microsoft Excel, version 2011 (Microsoft Corp., Redmond, WA, USA).

Any disagreements on paper inclusion and data extraction were resolved by a third investigator (A.I.). No search was performed to retrieve any unpublished data or abstracts. Studies that had reported evidence of bias of insufficient data were excluded.

The International Prospective Register of Systematic Reviews (PROSPERO) was referred to to avoid duplication, and the full protocol of this study was registered (ID1041579).

### 2.2. Outcomes of Interest, Data Extraction and Assessment of Study Quality and Risk of Bias

We aimed to analyze the current treatment option for aberrant visceral artery aneurysm and the related outcomes (mortality, bleeding, end-organ ischemia, lesions of the surrounding organ, need for reintervention) to investigate the potential pitfalls reported during treatment and help in informing the selection of each interventional treatment, based on individual procedural risks in future clinical practice.

Therefore, the following data were retrieved from the included papers in a predefined database: authors, year of publication, study design, number of included patients, demographics (age and sex), type of treatment performed, early and long-term mortality, end-organ ischemia, complications, reintervention rate and follow-up length. The need for and type of additional procedures and unexpected maneuvers performed during the elected treatment were also narratively reported to instruct physicians in terms of future management.

As there were no randomized studies, or clinical trials, case reports and case series were assessed according to the CARE guidelines and the Joanna Briggs Institute (JBI) Critical Appraisal Checklist for case reports/studies and case series in terms of design, heterogeneity, and possible bias [9,10].

### 2.3. Statistical Analysis

Data synthesis was conducted independently by two independent investigators (A.L. and G.P.). All data for an observation that had one or more missing values were deleted, and the analysis was run only on observations that had a complete set of data.

SPSS statistical software version 22 (IBM Corporation, Armonk, NY). was used for analysis. Because the number of cases was too small and reported data too heterogenous, no meta-analysis for measuring the pooled clinical results was performed.

The extracted data are reported in percentages and absolute values or synthetized narratively.

## 3. Results

### 3.1. Demographics and Clinical Data

Overall, 37 papers were screened (Figure 1), and 30 papers (27 about true aneurysms and 3 reporting pseudoaneurysms of aberrant visceral arteries) were included in the analysis, for a total of 36 patients (25/36, 69.4% female) (Table 1). The mean age at presentation was 48.9 ± 12.8 years, range 22–73 years.

Most of the included patients had true aneurysms (33/36, 91.7%), whereas 3/36 (8.3%) had pseudoaneurysms. Aneurysm diameter was reported in 34/36 patients, with a mean size of 30.5 mm (range 6–60 mm); in two cases, aneurysm size was not specified.

Clinical presentation was described for all included patients. Overall, 26/36 (72.2%) were symptomatic, most commonly with abdominal or epigastric pain, nausea or vomiting, back pain, or shortness of breath. Nine patients (25%) presented with acute abdominal or thoracic pain related to aneurysm rupture. The remaining 10/36 (27.8%) aneurysms were discovered incidentally in asymptomatic patients (Table 2).

Ethnicity was reported in only six cases (including two Asian, three Caucasian and one American patient), and comorbidities and the setting of aneurysm development were inconsistently described across included studies, preventing the identification of any reliable etiologic pattern.

### 3.2. Anatomical Variation

Lesions most frequently involved an aberrant splenic artery (30/36, 83.3%). Among these, the most common anatomical variant was a splenic artery originating from the superior mesenteric artery, while three patients (3/36, 8.3%) presented a common splenomesenteric trunk, and one patient (1/36, 2.8%) had a superior polar artery arising as an aberrant branch from the splenic hilum.

The aneurysmal involvement of other aberrant visceral arteries was less common. One lesion affected the gastroduodenal artery originating directly from the aorta (1/36, 2.8%).

Hepatic arterial variants included one right hepatic artery originating from the superior mesenteric artery (1/36, 2.8%), one accessory left hepatic artery arising from the left gastric artery (1/36, 2.8%), and one case of a right hepatic artery aneurysm associated with an aberrant left hepatic artery (1/36, 2.8%). Additionally, a celiomesenteric trunk with the aneurysmal involvement of the superior mesenteric artery was reported in one patient (1/36, 2.8%).

### 3.3. Interventional Setting and Strategies

The setting for repair was described in 35 cases: 4/36 (11.1%) were managed as urgent presentations and 31/36 (86.1%) as non-urgent, and 1/36 (2.8%) was not specified.

Treatment strategies were heterogeneous but mostly minimally invasive. Overall, 19/36 (52.8%) patients underwent purely endovascular repair, 15/36 (41.7%) were treated with open surgery, and 2/36 (5.6%) had hybrid procedures combining endovascular coiling with laparoscopic splenic artery ligation. Details are provided below:

1. Endovascular approaches included coil embolization alone (7), covered stent placement (1), combined coiling of the lesion and stenting (9) or Amplatzer plug (Abbott Laboratories, Chicago, IL, USA) positioning (2) and distal splenic artery and aneurysm sac coiling with SMA stenting (1).
2. Open procedures (which were carried out in 15/36 patients, 41.7%; mean aneurysm diameter 34.3 mm, range 18–60) included aneurysmectomy with splenic artery ligation (3 patients), aneurysmectomy with SMA repair or bypass, laparotomic aneurysmorrhaphy without revascularization, median arcuate ligament release without direct aneurysm repair, and various reconstructions such as splenic artery reimplantation into the SMA or primary SA–SMA.
3. Hybrid treatment, consisting of coil embolization followed by laparoscopic ligation of the distal splenic artery, was reported in two patients (5.6%).

### 3.4. Outcomes

Early outcomes were favorable. A single organ-related complication was reported (1/36, 2.8%), consisting of mild pancreatitis following hybrid treatment consisting of coiling and laparoscopic distal SA clipping, which resolved with conservative management. No perioperative or aneurysm-related deaths were reported in any of the included cases (Table 3).

Local complications occurred in a patient who experienced postoperative bleeding seven days after laparotomic aneurysmorrhaphy, requiring reintervention for definitive hemostasis. No further access site or wound-related complications were described.

Endovascular procedures demonstrated a high rate of technical success; however, in two cases, the persistent retrograde perfusion of the aneurysm sac required hybrid completion with laparoscopic distal splenic artery ligation.

Follow-up information was available for 28/36 patients, whereas it was not reported for eight. Among those with available data, the median follow-up was 6.0 months (mean ≈ 10.5 months, range 1.5–42 months). No recurrent aneurysms or late aneurysm-related complications were described during the reported follow-up intervals.

## 4. Discussion

According to reported data on aneurysms affecting non-aberrant visceral arteries, these lesions are mostly asymptomatic, and hence diagnosis is often incidental on abdominal images (ultrasound—US—or Computed Tomography Angiography—CTA) performed for other conditions. Despite being extremely rare (20% of cases in general population) [2], prognosis upon rupture, which may represent the first sign of the lesion, is high (reaching 5–25% of reported cases), making these conditions clinically relevant [41,42].

This review demonstrated that most reported cases (72%) were symptomatic upon initial presentation, making this aneurysm potentially more insidious as it is more frequently treated on an urgent basis.

Several factors are associated with the development of aneurysm affecting visceral arteries (including atherosclerosis, fibrodysplasia, portal hypertension, vasculitis, abdominal trauma, or iatrogenic injuries), and they are not different for aberrant vessel lesions [43].

Anatomically, those affecting the SA and a common HA are the most frequent [2].

The SA variation most frequently encountered is a spleen–mesenteric trunk in which the SA arises from the superior mesenteric artery (1% of the general population) [44,45].

**Figure 2:**
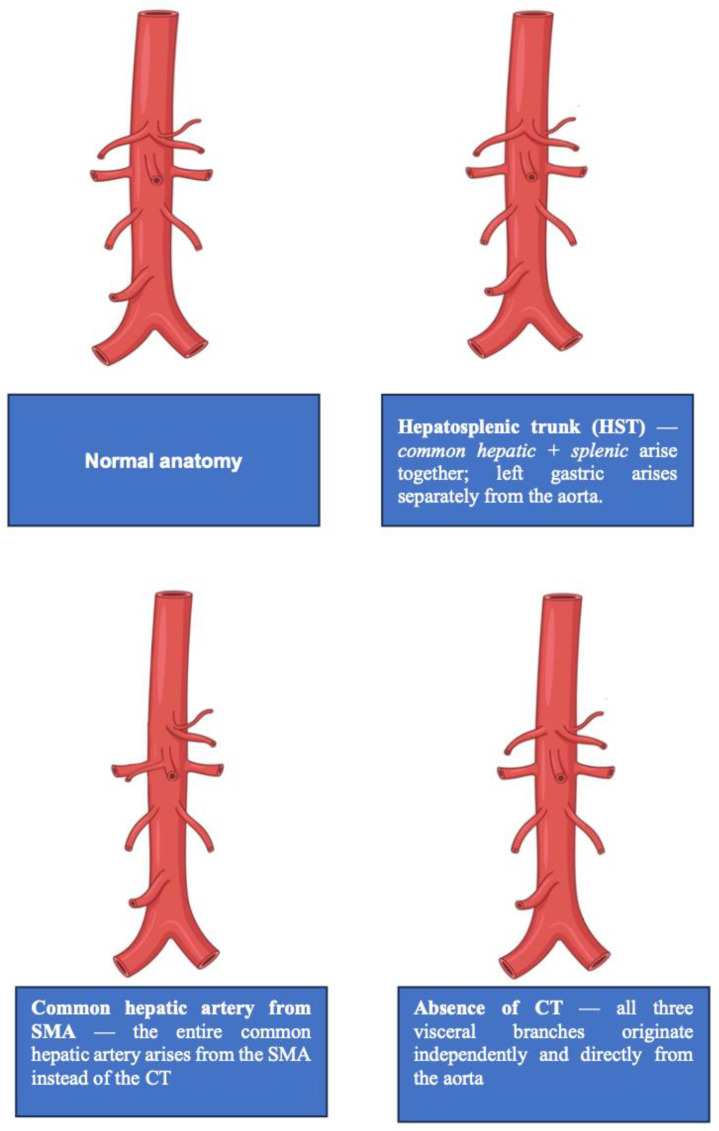
Most frequent variation in origin of visceral vessels.

Regarding aberrant HAs, overall, 13–60% originate from the SMA, but a right HA arising from the gastroduodenal artery may also be encountered. This variation should always be recognized as it is associated with an increased risk of hemorrhagic and ischemic complications during laparotomy for abdominal disease [46] (Figure 2).

The diagnosis of the anomalous origin of the visceral vessels is frequently incidental, but when an intervention is planned, anatomical variation should be attentively considered. Arteriography, CTA or Magnetic Resonance Angiography (MRA) is indicated to study the anatomical variation and to detect associated aneurysms in other districts [4,47].

Multidisciplinary imaging review (vascular surgery, interventional radiology, and hepato-pancreatobiliary or gastrointestinal surgery as relevant) improves planning and reduces surprises in the operating room or inappropriate treatment choices [47].

Specific guidelines for the management of aberrant visceral aneurysm are lacking, and treatment criteria mirror those applied to visceral aneurysms in the absence of anatomical variations.

With regard to treatment, aneurysms arising from aberrant visceral arteries present unique challenges because their origin, course, and collateral networks deviate from standard anatomy, and end-organ ischemia or infarction due to embolization or the inadvertent occlusion of an aberrant artery supplying a major organ is a major concern when the aberrant vessel is the dominant supply. Although this represents a review of individual case reports and may provide only limited technical details, several procedural challenges can be inferred from the therapeutic strategies described in this paper in the analysis.

Overall, contemporary practice favors endovascular repair for many visceral artery aneurysms due to lower perioperative morbidity. Success depends on vessel tortuosity, landing zones, and the presence of important side branches that supply end organs. Embolization is the most commonly used approach despite the risk of mesenteric ischemia from coil migration, which should not be understated [4,48,49]. Coil embolization frequently requires the embolization of both the aneurysm sac and the proximal/distal artery, reflecting the risk of non-target embolization and persistent retrograde inflow; stent graft placement may also present several challenges, including the need for precise landing zones in short aberrant branches and the risk of covering adjacent visceral branches. Tortuous or small-caliber aberrant vessels increase the likelihood of failed endovascular access, device malposition, endoleak, or the need for open conversion, with attendant higher perioperative risk [2,4,48,49].

Overall, when the aberrant artery is nonessential or has robust collaterals, selective embolization may be acceptable; when it is dominant, revascularization, bypass, or open repair should be considered to avoid ischemia.

Open repair remains necessary in select cases—for example, when the aneurysm involves multiple branching points or when endovascular access is not feasible—but carries higher immediate morbidity and requires careful planning to preserve perfusion, and the technical difficulty of obtaining safe proximal and distal vascular control in aberrant arteries should not be underestimated [4,47]. Organ perfusion monitoring, selective balloon occlusion tests, and staged embolization can reduce ischemic complications but do not seem to represent the standard of care [4].

A surgical approach is also normally planned in fit patients due to anatomical restriction for efficacious endovascular management. This is, for instance, the case of an anomalous SA characterized by a short aneurysmal neck or an SA close to the SMA origin. Open resection might be more appropriate, allowing for aneurysmorrhaphy, ligation or aneurysmectomy associated or not with revascularization with surgical bypass according to the status of the collateral network from short gastric vessels [19,21,23,26,29].

Splenectomy may be performed, but the individual risk of infection should be discussed preoperatively. This may be indicated for aneurysm affecting the proximal third of the SA [35].

Overall, technical pitfalls include underestimating the need for branch preservation; the embolization/surgical ligation of an aberrant artery without confirming collateral supply may lead to organ ischemia.

Patient selection and detailed anatomic mapping are decisive as inadequate imaging or failure to recognize an aberrant origin can lead to the incomplete exclusion or inadvertent sacrifice of critical branches. Vascular surgery, interventional radiology, hepatobiliary/visceral surgery, and anesthesiology should jointly be used to decide on approach (endovascular vs. open vs. hybrid) for aberrant vessels [4,47].

## 5. Conclusions

Aneurysm and pseudoaneurysm affecting aberrant visceral arteries are uncommon but represent a potentially severe disease.

An interventional approach should be taken to prevent the risk of rupture according to the features and size of the lesion, for which a preoperative anatomical study is mandatory to customize the therapeutic strategy.

Meticulous preoperative imaging, multidisciplinary planning, and individualized modality selection are essential to avoid serious pitfalls such as organ ischemia, incomplete exclusion, and device-related complications.

Where anatomy permits, endovascular approaches offer lower perioperative risk, but the preservation of end-organ perfusion must never be sacrificed for technical simplicity; when uncertainty exists, open repair or hybrid strategies remain valid options and should be guided by current guidelines and expert consensus.

Understanding the anatomy of visceral arteries and their variations is paramount in clinical practice to minimize procedural risks, optimize outcomes, and prevent potential complications.

## 6. Limitation

This study is inherently limited by the non-standardized nature of case reports that were selected for the analysis, impacting the uniformity of the variables of interest. This review has several limitations. The overall number of available studies was small, with limited sample sizes and no randomized controlled trials. Long-term follow-ups were inconsistently reported. Due to this variability and the low number of cases, it was not possible to perform pooled statistical analysis or meta-analysis, but a narrative synthesis was instead performed.

## Figures and Tables

**Table 1:** Overview of included studies.

Authors	Article Type and Year	Age and Gender	Type of Aberrant Vessel Origin	Type of Lesion and Size (Maximal Diameter)	Type of Treatment and Outcomes	Follow-Up Length
Ghatan et al. [11]	Case report, 1967	58 y; Female	SA from SMA	Aneurysm, 40 mm	Open aneurysmectomy + SMA primary repair + splenectomy	NR
Sidhu et al. [12]	Case report, 1995	35 y; Female	SA from SMA	Aneurysm, 20 mm	Open aneurysmectomy	NR
Settembrini et al. [13]	Case report (2 cases), 1996	45 y; Male 43 y; Female	(1) SA from SMA (2) Common splenomesenteric trunk	Aneurysm, (1) 40 mm (2) 32 mm	(1) Open aneurysmectomy + SMA repair + splenic artery ligation + splenectomy (2) Open aneurysmectomy with preservation of SMA and splenic artery	(1) 12 months (2) 12 months
Patel et al. [14]	Case report, 1998	59 y; Female	SA from SMA	Aneurysm, 50 mm	Open aneurysm excision + SMA–splenic artery bypass using reversed saphenous vein graft	6 months
Feo et al. [15]	Case report, 2004	64 y; Male	SA from SMA	Aneurysm, 43 mm	Open aneurysmectomy + splenic artery reimplantation into SMA	3 months
Migliara et al. [16]	Case report (2 cases), 2005	50 y; Female 47 y; Male	SA from SMA	Aneurysm, 25 mm	(1) Open aneurysmectomy + splenic artery ligation (no reconstruction, spleen preserved) Endovascular coil embolization + proximal/distal SA occlusion	(1) 10 days (2) NR
Mastracci et al. [17]	Case report (2 cases), 2005	31 y; Female 42 y; Female	SA from SMA	Aneurysm, 27 mm and 33 mm	(1) Coil + laparoscopic splenic artery clipping (distal to aneurysm)	9 months
					(2) Coil + laparoscopic distal SA clipping	8 months
Sato et al. [18]	Case report, 2006	50 y; Female	Common splenomesenteric trunk	Aneurysm, 25 mm	Coil embolization	22 months
LaBella et al. [19]	Case report, 2006	29 y; Female	SA from SMA	Aneurysm, 30 mm	Open ligation of splenic artery aneurysm (medial visceral rotation)	NR
Facy et al. [20]	Case report, 2006	36 y; Male	SA from SMA	Aneurysm, 34 mm	Open surgery—endoaneurysmorrhaphy (resection without reconstruction) + splenic preservation	6 months
Illuminati et al. [21]	Case report, 2007	51 y; Female	SA from SMA	Aneurysm, 20 mm	Open aneurysmectomy + SMA lateral repair + distal splenic artery ligation (spleen preserved)	6 months
Tanigawa et al. [22]	Case report, 2009	45 y; Male	SA from SMA	Aneurysm, 34 mm	Coil embolization	3 months
De Cloedt et al. [23]	Case report, 2010	41 y; Female	SA from SMA	Aneurysm, 23 mm	Open aneurysmectomy + proximal/distal ligation, no reconstruction, spleen preserved	1.5 months
Taneja, M et al. [24]	Case report, 2011	34 y; Male	SA from SMA	Aneurysm, 24 mm	Stenting (Advanta V 12 7 × 22 mm + 7 × 40 mm per EL IA)	6 months
Jiang et al. [25]	Case report, 2011	67 y; Female	SA from SMA	Aneurysm, 21 mm	Coil (distal SA + aneurysm sac) embolization + SMA stenting (10 × 40 mm Fluency stent graft)	12 months
Wong et al. [26]	Case report, 2013	40 y; Female	SA from SMA	Aneurysm, 26 mm	Open aneurysmectomy + primary SA–SMA anastomosis (spleen preserved)	NR
Kulkarni et al. [27]	Case report, 2013	35 y; Male	SA from SMA	Aneurysm, 22 mm	Coil embolization + stent 8 mm × 4 cm Fluency Plus	36 months
Zhou et al. [28]	Case report (4 cases), 2014	37 y; Male 36 y; Female 73 y; Male 52 y; Male	SA from SMA	Aneurysm, 40 mm Aneurysm, 36 mm Aneurysm, 59 mm Aneurysm, 35 mm	(1) Coil embolization of SA + covered stent in SMA (2) Coil embolization + stenting (10 mm × 40 mm, Wallgraft) (3) Coil embolization + stenting (10 mm × 50 mm, Wallgraft) (4) Coil embolization + stenting (10 mm × 50 mm, Wallgraft)	(1) 12 months (2) 12 months (3) 6 months (4) 10 months
Shukuzawa et al. [29]	Case report, 2015	63 y; Female	Right HA aneurysm with aberrant left HA	Aneurysm, 60 mm	Laparotomic aneurysmorrhaphy without revascularization (reopened after 7 days due to bleeding)	3 months
Jayakuman et al. [30]	Case report, 2017	22 y; Female	SA from SMA	Aneurysm, 30 mm	Amplatzer plug (distal) + covered SMA stent (inflow exclusion)	NR
Grippi et al. [31]	Case report, 2018	73 y; Female	SA (polar artery) as aberrant branch arising from splenic hilum	Pseudoaneurysm, 6 mm	Coil embolization	4 months
Qiu et al. [32]	Case report, 2019	45 y; Female	SA from SMA	Aneurysm, 29 mm	Distal SA + aneurysm sac coil embolization + SMA covered stent	6 months
Borioni et al. [33]	Case report, 2019	53 y; Female	SA from SMA	Aneurysm, 20 mm	Implantation of multiple coils and Amplatzer vascular plug	2 months
Serena et al. [34]	Case report, 2020	71 y; Female	Left accessory HA originating from left gastric artery	Pseudoaneurysm	Coil embolization	NR
Kakamada et al. [35]	Case report, 2021	52 y; Female	Common splenomesenteric trunk	Aneurysm, 45 mm	Aneurysmectomy and SA ligation	4 months
Shreshta et al. [36]	Case report, 2021	45 y; Female	Gastroduodenal artery originating directly from aorta	Pseudoaneurysm	Coil embolization	NR
Ichikawa et al. [37]	Case report, 2022	63 y; Male	SA from SMA	Aneurysm, 22 mm	Coil embolization	36 months
Kuwada et al. [38]	Case report, 2022	62 y; Female	SA from SMA	Aneurysm, 22 mm	Coil embolization + stenting (Viabahn, 7 × 25 mm and 8 × 25 mm)	42 months
Braet et al. [39]	Case report, 2023	50 y; Female	SMA aneurysm associated with CMT	Aneurysm, 18 mm	Open median arcuate ligament (MAL) release only, no direct aneurysm repair	6 months
Çildağ et al. [40]	Case report, 2023	60 y; Female	Right HA originating from SMA	Aneurysm, 50 mm	Coil embolization + stenting (7 × 38 Atrium V12 Advanta)	6 months

NR—not reported; SA—splenic artery; SMA—superior mesenteric artery; HA—hepatic artery; EL—endoleak; CMT—celiacomesenteric trunk.

**Table 2:** Summary of baseline data of population included in analysis.

Demographics and Clinical Data	
	N (%)
Sex	11 male (30.6%) 25 female (69.4%)
Age	median age 50 years (range 22–73 years)
Type of lesion	
True aneurysm	33 (91.7%)
Pseudoaneurysm	3 (8.3%)
Mean aneurysm diameter	30.5 mm (range 6–60 mm)
Affected artery	
Aberrant splenic artery	27 (75.0%)
Aberrant hepatic artery	3 (8.3%)
Aberrant superior mesenteric artery	1 (2.8%)
Aberrant gastroduodenal artery	1 (2.8%)
Branch of aberrant splenic artery	1 (2.8%)
Celiomesenteric trunk involvement	1 (2.8%)
Main comorbidities	
Osler–Weber–Rendu syndrome	1 (2.8%)
Systemic lupus erythematosus	1 (2.8%)
Clinical presentation	
Chronic abdominal/back pain	12 (33.3%)
Asymptomatic	10 (27.8%)
Regurgitation and eructation	1 (2.8%)
Sepsis	1 (2.8%)
Claudicatio abdominis	1 (2.8%)
Acute abdominal/thoracic pain	9 (25.0%)

**Table 3:** Treatment strategies and outcomes.

Treatment and Outcomes	
	N (%)
Setting	
Elective	31 (86.1%)
Emergent/urgent	4 (11.1%)
Open repair	15 (41.7%)
Aneurysmectomy	10 (27.8%)
Ligation	2 (5.6%)
Aneurysmorrhaphy	3 (8.3%)
Endovascular repair	19 (52.8%)
Embolization with only coils	6 (16.7%)
Embolization with multiple agents	3 (8.3%)
Stent grafting	1 (2.8%)
Assisted coiling (stent graft + coiling)	9 (25.0%)
Hybrid repair (Coiling and laparoscopic ligation)	2 (5.6%)
Other/treatment refusal	2 (5.6%)
Outcomes	
Aneurysm rupture	2 (5.6%)
End-organ ischemia	2 (5.6%)
Local complications	1 (2.8%)
Mortality	-
Reintervention	1 (2.8%)
Mean follow-up	10.5 months (range 10 days–42 months)

## Data Availability

No new data were created or analyzed in this study.

## Supplementary Materials

The following supporting information can be downloaded at https://www.mdpi.com/article/10.3390/jpm16030165/s1, Table S1: PRISMA 2020 checklist [8]; Table S2: PICO framework.

## Abbreviations

The following abbreviations are used in this manuscript:

HA	hepatic artery
SMA	superior mesenteric artery
SA	splenic artery
US	ultrasound
CTA	computed tomography angiography
MRA	magnetic resonance angiography
EL	endoleak
CMT	celiacomesenteric trunk
