# Supplementary material for: Tailored Interventional Approaches to the Management of True and False Aneurysms Affecting Aberrant Visceral Arteries Are Associated with Enhanced Clinical Outcomes
Source: J Pers Med. 2026 Mar 16;16(3):165. doi: 10.3390/jpm16030165 (PMC13028169; doi:10.3390/jpm16030165)
Supplement: Supplementary file 1 [file jpm-16-00165-s001.zip › jpm-4114677-Supplementary Table S2-PICO framework.pdf]

Supplementary Table S2. PICO framework

PICO ELEMENTS	Keywords	Search terms
**P (Patient or Population)**	Patients affected with aneurysm or pseudoaneurysm involving aberrant visceral arteries	(“aberrant” OR “anomalous” OR “ectopic” OR “abnormal” OR “variant” OR “accessory”) AND (“artery") AND (“visceral” OR “splanchnic” OR “celiac” OR “mesenteric” OR “hepatic” OR “splenic” OR “renal” OR “gastric” OR “pancreatic” OR “gastroduodenal” OR “ileocolic” OR “jejunal” OR “colic” OR “phrenic” OR “suprarenal” OR “middle colic” OR “pancreaticoduodenal”) AND ("aneurysm" OR "dilation" OR "vascular dilation" OR "bulge" OR "sac" OR "ectasia" OR "true aneurysm" OR "pseudoaneurysm" OR "false aneurysm" OR " hematoma" OR "rupture" OR "burst" OR "tear" OR "perforation" OR "laceration")
**I(Intervention)**	Endovascular treatment of aneurysm and pseudoaneurysm affecting aberrant visceral arteries	“Treatment” OR “Endovascular” OR “Embolization” AND “Repair” OR “Management” OR “Intervention” OR “Procedure” OR “Technique” OR “Approach” OR "Minimally invasive" OR “Reconstruction” OR "Percutaneous treatment
**C(Comparison)**	Open treatment of aneurysm and pseudoaneurysm affecting aberrant visceral arteries	“Surgical” AND “Repair” OR “Management” OR “Intervention” OR “Procedure” OR “Technique” OR “Approach” OR "Open surgery"
**O (outcomes)**	Ischemic complication for end-organs mortality	“Death” OR “Mortality” OR “Survival” OR “Complication” OR “Visceral” OR “Mesenteric” AND “Ischaemia”
